# Targeting the Notch receptor dimerization domain to inhibit Notch signalling—A new avenue of therapeutics

**DOI:** 10.1111/febs.70423

**Published:** 2026-01-28

**Authors:** Gerard F Hoyne

**Affiliations:** ^1^ School of Health Sciences The University of Note Dame Australia Fremantle Australia; ^2^ The Institute for Respiratory Health, Harry Perkins Medical Research Institute Nedlands Australia

**Keywords:** cancer, dimerization, immunity, Notch receptor, proteolysis

## Abstract

Notch signalling is an evolutionarily conserved signalling pathway that directs cell growth and differentiation across multiple tissue types, and its regulation must be controlled across the lifespan. Aberrant Notch signalling due to genetic mutations that occur within the negative regulatory region of the Notch 1 gene is linked to the development of acute T‐cell leukaemia in humans. This discovery has led to a concerted effort to understand how Notch receptor signalling is regulated in mammalian cells. Liu *et al.* have developed a range of novel peptide inhibitors that target the heterodimerization domain within the negative regulatory region of the Notch receptor. They show that the peptide inhibitors are specific to a Notch receptor paralogue. The possible biological and therapeutic consequences are discussed.

## Graphical abstract

Notch signalling is an evolutionarily conserved signalling pathway that directs cell growth and differentiation across multiple tissue types, and its regulation must be controlled across the lifespan. Aberrant Notch signalling due to genetic mutations that occur within the negative regulatory region of the Notch 1 gene is linked to the development of acute T‐cell leukaemia in humans. Liu *et al.* developed a range of novel peptide inhibitors that target the heterodimerization domain within the negative regulatory region of the Notch receptor. Here, the possible biological and therapeutic consequences are discussed.

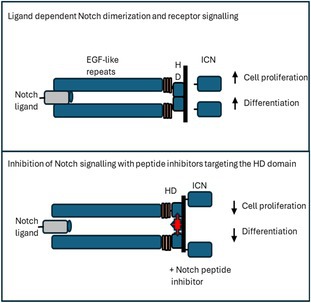

Comment on: https://doi.org/10.1111/febs.70312.

**Abbreviations**
DAPT: N‐(N‐(3,5‐difluorophenacetyl)alanyl)phenylglycine tert‐butyl ester
Dll: Delta‐like
EGF: epidermal growth factor
GSIs: gamma secretase inhibitors
HD: heterodimerization domain, LNR, Lin12, Notch repeats
ICN: intracellular domain of Notch receptor
Jag: Jagged
NRR: Notch regulatory domain
T‐ALL: T‐cell acute lymphocytic leukaemia
Th: T helper cell

## Introduction

**Fig. 1:**
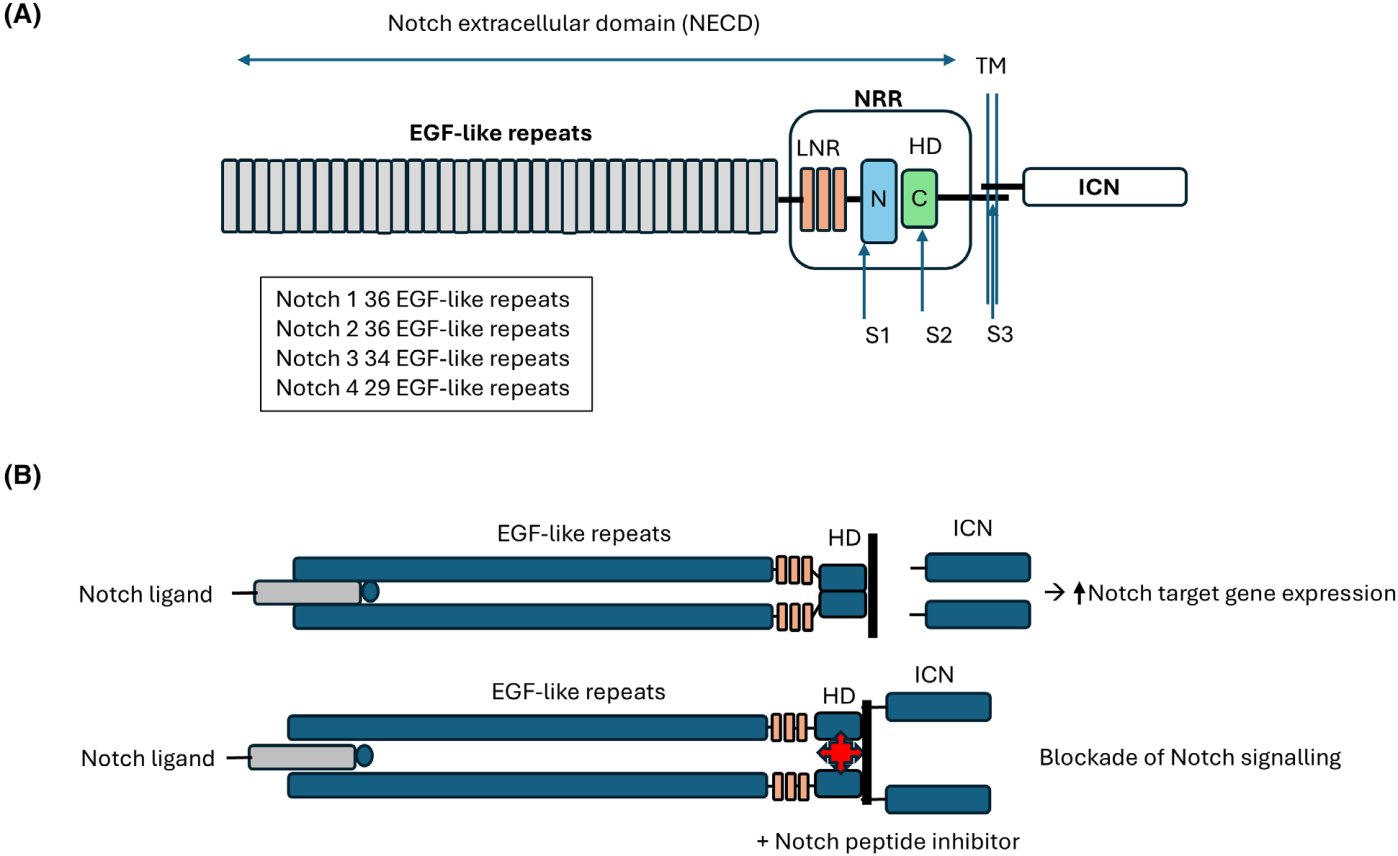
Structural domains and signalling of Notch receptors. (A) The Notch receptor has an extracellular domain composed of a variable number of EGF‐like repeats and the Notch regulatory region (NRR) is comprised of three Lin12/Notch repeats (LNR) and the heterodimerization (HD). The receptor has a transmembrane and intracellular domain (ICN) which is responsible for signal transduction and target gene expression. Upon binding of a Notch ligand, the receptor undergoes a series of proteolytic cleavage events. The HD region of the NRR is cleaved by an ADAM protease at the S2 site and followed by a presenilin‐dependent gamma secretase cleavage at the S3 site that releases the ICN from the membrane. (B) As described by Liu et al. [6] the HD domain contains a conserved helical structure that plays an important role in receptor dimerization and signalling. The HD region can be targeted by Notch paralogue‐specific peptide inhibitors to block receptor dimerization and Notch signalling.

The Notch receptor family is part of an evolutionary conserved signalling pathway that plays an important role in embryonic development and tissue homeostasis in metazoan animals. Mammals have four Notch receptors (Notch 1–4) and five ligands Delta‐like (Dll)‐1, Dll3, Dll4 and Jagged (Jag)‐1 and Jag‐2 [1]. Notch receptors are type 1 transmembrane receptors with the extracellular domain composed of a variable number epidermal growth factor (EGF)‐like repeats, depending on the Notch allele, a heterodimerization (HD) domain and transmembrane domain. Structural studies have highlighted the importance of the HD domain that has three Lin‐12/Notch repeats and a negative regulatory region (NRR) that is positioned close to the TM region [2, 3] (Fig. 1). The NRR region on the Notch receptor is composed of conserved helices that form an interface to reinforce the autoinhibitory structure of the Notch receptor that precludes exposure of an ADAM metalloprotease S2 cleavage site within this domain [3]. Binding of Notch ligand to its receptor leads to a conformational change to the NRR exposing the S2 cleavage site that is cleaved by an ADAM‐type protease and allows a subsequent cleavage of the S3 site by gamma secretase [4, 5]. The ligand‐induced proteolytic events lead to the release of the intracellular domain that translocates to the nucleus to drive Notch target gene expression [1].

Dimerization of Notch receptors represents a critical step in mediating downstream signalling events that is linked to cell growth and differentiation. Therapeutic strategies to date have relied on the use of gamma secretase inhibitors (GSIs), which have been the mainstay in clinical medicine for the treatment of T‐cell leukaemias and other cancers. However, a major limitation on the use of GSIs relates to their toxicity. The development of small peptide inhibitors that could target specific Notch receptors to render them inactive could offer a novel targeted precision medicine approach that could benefit a range of human diseases.

## Identification of the NRR for Notch receptor dimerization

The study by Liu *et al*. reported in *The FEBS Journal* [6] showed that the NRR is conserved across the different Notch receptors and contains a core helical motif. Disruption of the helical motif could abrogate Notch receptor dimerization and blocked stable ligand‐Notch receptor dimerization at the cell surface. Introduction of point mutations into the NRR region of the Notch 3 receptor identified a key target sequence that could block ligand‐dependent Notch 3 cleavage and transactivation of a Notch3 reporter gene. The NRR dimerization motif was required for activation of downstream Notch target genes of the Hairy Enhancer of Split complex (e.g. Hey1 and Hes1).

The conservation of the NRR region across the four Notch receptors prompted Liu et al. to design and test whether specific peptides could target the HD domain interface to interfere with Notch 3 dimerization and downstream signalling. Whilst the Notch 3 peptide inhibitor could block Notch 3 receptor activity, it had no effect on either Notch 1 or Notch 2 receptor dimerization and cleavage or downstream signalling. This led the investigators to design Notch paralogue‐specific peptide inhibitors. They found that the Notch1 peptide inhibitors could block Notch 1 but not Notch 2 or 3, and likewise, the Notch 2 peptide inhibitor was specific for inhibition of Notch 2 dimerization and signalling only.

To investigate the potential clinical relevance of the NRR peptide inhibitors, they examined six different patient cell lines; four of these had Notch 1‐activating mutations, and two cell lines acted as controls that either lacked expression of the Notch 1 extracellular domain (i.e. lacked the NRR region) or expressed a Notch1 receptor that was not hyperactivated. They compared the efficacy of the Notch1 inhibitor peptides against the DAPT GSI against the various cell lines. These studies revealed that the Notch1 peptide inhibitors could suppress cellular proliferation of the four cell lines with activated Notch receptors but did not affect growth of the control cells. Finally, they used a zebrafish model to show that the Notch1 peptide inhibitors could block tissue invasion by human T‐ALL cells harbouring activated Notch1 mutations.

A limitation of the current study was that the authors focused on the use of *in vitro* cell lines harbouring Notch1 mutations. T‐ALL is a relatively common malignant haematopoietic disease in humans, and cell lines have been readily established and characterized. However, it remains to be seen whether the Notch paralogue‐specific peptide inhibitors can modulate Notch‐mediated diseases *in vivo*.

## Assessing modulation of Notch signalling in immune‐mediated diseases

In addition to its role in T‐cell leukaemia and cancers, Notch signalling has critical roles in different immune cell subsets over the human life span [1, 7]. Notch receptors and ligands have been studied in T‐ and B‐cell development [8, 9] and in adaptive immune function, especially for regulatory T cells and conventional T‐cell responses that impact CD4+ and CD8+ T cells [10, 11, 12]. For example, Notch1/Jagged1,2 and Delta1, four interactions can bias Th cell functions [11]. CD4+ T helper 2 responses drive allergic disease, and various allergen‐specific animal models have been developed. It would be interesting to see how these new Notch paralogue peptide inhibitors might function in modulating immune‐mediated diseases in preclinical *in vivo* mouse models.

## Conclusion

The study by Liu *et al*. provides further insight into the role of the NRR of Notch receptors. Through the specific actions of Notch paralogue peptide inhibitors, it may be possible to modulate the activity of specific Notch receptors involved in a disease process whilst sparing the other Notch receptors to perform their normal physiological roles.

## Conflict of interest

The author declare no conflict of interest.
